# Assessing the Risk Factors for Refractory Eosinophilic Esophagitis in Children and Adults

**DOI:** 10.1155/2019/1654543

**Published:** 2019-01-13

**Authors:** Erminia Ridolo, Irene Martignago, Irene Pellicelli, Cristoforo Incorvaia

**Affiliations:** ^1^Department of Medicine and Surgery, University of Parma, Parma, Italy; ^2^UOSD Allergology, ULSS 1 Dolomiti, S. Maria del Prato Hospital, Feltre, Italy; ^3^Cardiac/Pulmonary Rehabilitation, ASST Pini/CTO, Milan, Italy

## Abstract

**Background:**

Up to one-third of the patients suffering from eosinophilic esophagitis (EoE) present a refractory form, as defined by nonresponsiveness in clinical, endoscopic, or histological assessment after first-line therapy. Several studies recently investigated which factors can influence the development of this disease, but very few analyzed the factors underlying refractory EoE.

**Methods:**

Medical charts of patients affected by EoE were retrospectively evaluated. Phenotyping of patients was conducted according to demographic, clinical, histological, and treatment variables. Then, patients were divided into responder and nonresponder to therapy and distinguished among children and adults.

**Results:**

Forty-five children and 35 adult EoE patients were included. In the pediatric population, female sex (*p* < 0.05) and a higher score of visual analogue scale (VAS) at the follow-up visit (*p* = 0.02) were significantly associated to the risk of refractory EoE. Among adults, statistical significance was reached for years of follow-up (*p* = 0.001), diagnostic delay (*p* = 0.03), use of antibiotics during infancy (*p* = 0.01), and food allergy (*p* = 0.04).

**Conclusions:**

Our study highlighted female sex and a higher VAS score at the time of follow-up visits as risk factors for refractory EoE in children, while the risk factors in adults were identified as fewer years of follow-up, greater diagnostic delay, use of antibiotics during infancy, and food allergy.

## 1. Introduction

Idiopathic eosinophilic esophagitis (EoE) is a chronic inflammatory antigen-mediated disease, characterized by a significant eosinophilic infiltration of the esophagus mucosa, with no other identifiable causes of local eosinophilia, and by an association with high risk of irreversible fibrosis, if left untreated [1]. EoE was recognized as a significant cause of morbidity in the last decades and nowadays is defined as “the most prevalent cause of chronic esophagitis after gastroesophageal reflux disease (GERD) and the leading cause of dysphagia and food impaction in children and young adults” [2]. In fact, EoE has an incidence of 1 in every 2000 inhabitants and a prevalence of 13 and 49 cases per 100,000 inhabitants [2, 3]. Any age can be concerned, even though diagnosis is more frequent during third/fourth decades [4]. Male sex is prevalently affected with a 3 : 1 ratio, likely because of the single-nucleotide polymorphism (SNP) of the thymic stromal lymphopoietin (TSLP) receptor located on chromosome Yp11.3 [5].

EoE pathogenesis is very complex and at the moment not completely clarified. However, both genetic and environmental factors play a fundamental role in the development of this pathology. Among genetic factors that explain an inheritance up to 70% [6], several altered molecules have been identified: eotaxin-3, TSLP and its receptor, filaggrin, desmoglein-1, calpain-14, and EMSY [7–9]. With regard to environmental factors, atopy represents the main comorbidity in patients affected by EoE, affecting up to 90% of both children and adults. In particular, both inhalant and food allergens seem to work as triggers for the Th2 inflammatory process with a possible IgE-mediated mechanism [2, 6]. Recent studies investigated also the role of IgG4 towards foods [10]. The main role in the pathogenesis of EoE is played by eosinophils, due to their ability to produce and activate important proinflammatory mediators, such as GM-CSF, TGF-*β*, TNF-*α*, Th2 lymphocytes, and their cytokines (IL-5, IL-4, and IL-13) [11]. Recently, a new promising role in etiopathogenesis has also been acknowledged for mast cells, invariant natural killer T (iNKT), and basophils [12, 13]. In the latest years, a small number of studies investigated the likely interplay among microbiome and development of EoE, with particular interest on the massive use of antibiotics during infancy [14].

Diagnosis of EoE is based on the combination of clinical symptoms and infiltration of esophageal mucosa by at least ≥ 15 eosinophils/high-powered fields (HPFs) [2]. Symptoms can vary, with a prevalence of food refusal, abdominal pain, and vomiting in infants/children and dysphagia, esophageal bolus, and retrosternal pain in adolescents/adults [15].

One of the most debated aspects of EoE is the treatment that consists of drugs (topical steroids and/or proton pump inhibitors), diet, and esophageal dilatation. Different approaches and combination of these treatments have been applied during the brief history of EoE, with variable results. The available data demonstrated that up to 30% of patients have a refractory disease [16]. The term refractory EoE, in fact, identifies those patients with EoE who are not responsive to therapy neither in clinical nor endoscopic or histological level. Currently, there are no reliable methods to identify patients at risk of refractory EoE.

The aim of this study was phenotyping of patients affected by EoE according to clinical and therapeutic variables and distinguishing responders and nonresponders to therapy among patients in pediatric and adult age, in particular by analyzing risk/protection factors for refractory EoE. Refractory EoE is an EoE that is not responsive to therapy neither in clinical nor endoscopic or histological level. We defined as refractory those patients that, after 8 weeks of first-step therapy (intended as dietetic therapy or topical corticosteroid therapy), still presented symptoms or histological signs. The second step consisted in the therapy not used as first line for at least another 8 weeks.

## 2. Methods

We performed a retrospective study on outpatients affected by EoE and visited at the Allergy and Clinical Immunology Clinic of the University Hospital of Parma between 2008 and 2016. Data analyzed were collected retrospectively from the paper and electronic medical records of patients. Study population was divided into group A (pediatric patients) and group B (adult patients), based on age at the time of diagnosis. Diagnosis of EoE was formulated according to consensus guidelines [2].

Patients in both groups were further divided, according to the response to therapy, into responsive EoE patients and refractory EoE patients. The following data were recorded for each patient and subsequently analyzed as variables of responsiveness or nonresponsiveness to therapy:

- Patient's sex
- Age at diagnosis
- Years of delay in diagnosis from the appearance of symptoms
- Symptoms at the onset of EoE
- Endoscopic findings at diagnosis
- Number of years of follow-up at the center
- Concomitant allergy to inhalants
- Concomitant food allergy (diagnosed by skin prick test and/or specific IgE assay according to current guidelines)
- Seasonality in the appearance of the symptoms of EoE
- Pharmacological/dietetic therapy at diagnosis
- Use of oral corticosteroids
- Need of endoscopic dilatation
- Therapy prescribed in case of flare-up
- Type of breastfeeding (maternal/artificial) during the first 6 months of life
- Repeated use of antibiotic therapy (intended as ≥3 cycles/year) in the first 3 years of life
- Smoking habit (only in the adult population)
- Subjective assessment of the state of well-being using the visual analogue scale (VAS) at the time of diagnosis and at every control visits that were scheduled every six months. The VAS implies ranges from a score of 0, corresponding to a state of complete well-being, to 10, corresponding to the worst state of malaise tolerable subjectively by the patient [17]

The characteristics of the examined populations are listed in Table 1 for group A and in Table 2 for group B.

The study was approved by the ethics committee of Parma (protocol number 44748).

### 2.1. Statistical Analysis

Statistical analysis was performed using the SAS 8.2 statistical software. The association between the detected variables and the outcome (nonresponsiveness to therapy) was evaluated by Pearson correlation. Multivariate regression analysis was, instead, used to test the independent effect among the different detected variables and the outcome of the study. *p* values lower than 0.05 were considered statistically significant.

## 3. Results

The results of the study are presented separately for the two populations. Group A included 45 subjects (30 males), with an average age at the moment of the diagnosis of 9.06 ± 4.31 years (range 2 to 17 years). Group B included 35 patients (28 males), with a mean age at the diagnosis of 36.5 ± 13.09 years (range 19 to 71 years).

### 3.1. Pediatric Population

In group A, patients with refractory EoE were 15 (8 males), with a mean age similar to that of responder subjects (8.93 vs. 9.13 years). With regard to sex, there were significantly more males than females among responders to treatment (22 males vs. 8 females, *p* < 0.05), while the number of nonresponder was comparable (8 males vs. 7 females).

Statistical significance was also detected for subjective assessment of the well-being status, through the use of the VAS scale at each subsequent follow-up visit. The achievement of a reduced level of VAS (on average 2.24 ± 1.94) in our study represents an index of protection with respect to possible relapses of the disease (*p* = 0.02).

The diagnostic delay, the years of follow-up, the clinical symptoms, and the endoscopic characteristics at the time of diagnosis as well as the concomitant allergy for food or inhalants, the seasonality of the symptoms, and the prescribed therapy (dietary or pharmacological) are not variable related to the risk of refractory EoE, according to our data. Similar results have been obtained for the use of antibiotics and maternal breastfeeding during infancy.

Data of group A are summarized in Tables 3 and 4.

### 3.2. Adult Population

In group B, patients with refractory EoE were 20 (16 males, mean age 39.4 years). Among responder and nonresponder, there were no significant differences as regards sex, age, clinical symptoms and endoscopic characteristics at the time of diagnosis, presence of sensitization to inhalants and seasonality in symptoms, prescribed therapy (dietary or pharmacological), maternal breastfeeding, smoking habit, and subjective assessment of well-being status through VAS scale at the time of diagnosis and at the following visits.

Analysis of the variables by Pearson correlation revealed a significant difference with regard to the years of follow-up and the use of antibiotics in childhood. Patients with refractory EoE were followed for a shorter time (3.0 years) than responder patients (4.66 years) with a significance equal to 0.001. The latter also reported less intensive use of antibiotic therapy during childhood (33.3%) compared to refractory EoE patients (70%) with a significance equal to 0.03 (Table 5).

Multivariate regression analysis confirmed the statistical significance for the use of antibiotics in childhood and also highlighted food allergy and delay in diagnosis as risk factors for the development of refractory EoE. In fact, among patients with food allergy, the 73.3% did not respond to therapy, with significant difference compared to responder subjects (*p* = 0.01). Regarding the diagnostic delay, an increase in the years required to diagnose EoE and initiate appropriate therapy correlates significantly with the risk of presenting refractory EoE (*p* = 0.04) in our subjects (Table 6).

## 4. Discussion

This study retrospectively evaluated patients affected by EoE referred at a single Italian Center of Allergy and Clinical Immunology. Patients' demographic data confirmed the findings reported in the literature: predominance in male sex, occurrence in both children and adults, and association between EoE and personal history of allergy, with frequent sensitization to inhalant and food allergens [11, 15, 18, 19]. With regard to incidence of refractory EoE, our data are also in line with those previously reported [16, 20], corresponding to one-third of all patients affected by EoE, at least in our pediatric population. Indeed, it was higher (57%) in our adult group.

There is a limited number of studies aimed at identifying the risk factors for the development of refractory EoE. The first study was conducted in 2004 on a small pediatric population (20 patients) by Noel et al., who identified atopy as the predictor of no response to topical corticosteroid therapy [21]. These data were confirmed by Konikoff et al. in 2006, again in a limited sample of children, 21 treated with swallowed fluticasone propionate and 15 treated with placebo [22]. More recent studies evaluated larger populations of patients. Leung et al., from the data obtained in 100 patients with refractory EoE and a mean age of 13 years, concluded that nonresponders had a higher eosinophil/HPF peak at the proximal and median esophagus compared with nonresponders [16]. Jensen et al. investigated in 127 children the influence of prenatal, intrapartum, and postnatal factors on the development of EoE. The results suggested that maternal fever during pregnancy, preterm labor, cesarean delivery, and antibiotic or acid suppressant use in infancy are risk factors for incurring EoE [23].

Another recent study analyzed the clinicopathologic and gene expression in biopsies from pediatric patients with refractory EoE. The authors highlighted that a small number of eosinophils/HPFs in the initial biopsy and the overexpression of RTNLB, a gene implicated in the remodeling of airways in asthmatic patients, are negatively correlated with the response to therapy in EoE [24].

All these studies considered nonresponders to topical steroid therapy and only the study by Konikoff et al. investigated also, as a second-line treatment, diet therapy and/or endoscopic dilatation [22]. In the study by Leung et al., analyzing the data from patients with refractory EoE on the second-line therapy, the authors found no significant difference between the variables considered (peak of eosinophils, age, sex, concomitant allergies, symptomatology at the onset, and endoscopic findings) [16].

Our study considered in particular pediatric patients who received topical steroid and/or dietetic therapy as first treatment. We identified female sex and a higher VAS at the time of follow-up visits as risk factors for refractory EoE. This finding could appear in contrast with the epidemiologic data on EoE, in which the male sex is far more affected. To date, however, there are no data that can confirm or deny our finding and certainly studies on larger populations are needed.

About the data of a higher VAS at the time of follow-up visits in patients with refractory EoE compared to responder subjects, we interpret this result as an indicator of poor or absent control of the disease. Indeed, flare-up of symptoms could have brought patients to an earlier follow-up visit, and this fact could be interfering with the consistence of our results.

With regard to adult patients with EoE, the few studies that analyzed possible risk factors for refractory disease reported data only concerning the response to topical steroid therapy. The first study was published in 2013 and identified as predictors of nonresponse three different variables: absence of episodes of esophageal bolus, advanced age, presence of signs of trachealization at the diagnosis, and the use of endoscopic dilatation [25]. Wolf et al. in 2016 confirmed that the need to perform an endoscopic dilatation at the diagnosis and, independently, abdominal pain as a symptom at the onset of EoE represented risk factors for refractory disease [20]. Similar results concerning endoscopic dilatation were also reported by Eluri et al. in the same year [26]. A more recent study analyzed the impact of smoking, alcohol consumption, and nonsteroidal anti-inflammatory drug (NSAID) use, concluding that NSAID and smoking appeared to be inversely associated with the risk of developing EoE. Refractory EoE was not investigated [27]. Analysis of our data from the group of adults identified a shorter period of follow-up and the diagnostic delay as risk factors for refractory EoE. In other words, a more recent but delayed diagnosis, compared to the onset of symptoms, was significantly related to the risk of failure to respond to EoE therapy. In fact, tissue fibrosis represents the natural evolution of EoE if left untreated, with a consequent increase in the risk of incurring in endoscopic dilatation. This aspect was reported as risk factor of refractory EoE in studies reported above and allows us to state that our findings are in line with data previously published.

Moreover, our data from adults highlighted that the use of antibiotics in childhood (intended as ≥3 cycles/year in the first 3 years of life) and food allergy are predictors of refractory EoE. The use of antibiotics during infancy has recently been correlated with the development of EoE [28], but as far as we know, it was never taken into consideration with regard to refractory EoE. Our study is then the first to investigate and highlight how frequent administration of antibiotics during infancy seems to correlate with resistance to therapy in case of EoE.

Atopy is a very common condition in patients with EoE. As reported above, positive history for allergy has been related to the lack of response to treatment in some pediatric cases, but it was never analyzed before in an adult population. This finding is of clear interest, considering that the role of food allergens in the pathogenesis and maintenance of EoE is not yet fully clarified.

Our study presents some limitations. First of all, it was a single-center study, and this can interfere with the generalizability of the findings. However, our populations had demographic and clinical characteristics comparable to those reported in the literature. Secondly, some data are self-reported by the patients, i.e., use of antibiotics during infancy, being unfeasible to confirm them in large part of the subjects. Anyway, similar studies on environmental or pre- and postnatal risk factors were concerned by the same limitation, due to the intrinsic nature of the collected data. On the other hand, the study also has some strengths. It was the first to analyze contemporarily a pediatric and an adult population, comparing responder to nonresponder patients with EoE. Moreover, some of the variables, i.e., the self-reported well-being status with VAS, have been analyzed for the first time in studies on refractory EoE.

## 5. Conclusion

Our study highlighted female sex and higher VAS at the time of follow-up visits as risk factors for refractory EoE in children and years of follow-up, diagnostic delay, use of antibiotics during infancy, and food allergy as risk factors in adults. Studies on larger populations are necessary to confirm our results, which, however, appear to be interesting in light of the lack of a definite elucidation of EoE pathogenesis.

## Figures and Tables

**Table 1:** Pediatric population characteristics (group A).

Variable	Responder patients (no. 30, 66.7%)	Refractory EoE patients (no. 15, 33.3%)
Age at the diagnosis (mean age)	9.13	8.93

Sex	22 M, 8 F	8 M, 7 F

Number of flare-ups (*n*, % pts)		1 = 4 pts
		2 = 5 pts
		3 = 5 pts
		4 = 1 pt

Diagnosis' delay from time of the symptom onset (mean in years)	1.48	2.03

Follow-up (mean in years)	5.18	4.73

Symptoms at the onset of EoE	Epigastric/abdominal pain = 12 pts	Dysphagia = 7 pts
	Vomit = 9 pts	Vomit = 6 pts
	Esophageal bolus = 8 pts	Esophageal bolus = 4 pts
	Dysphagia = 7 pts	Epigastric/abdominal pain = 4 pts
	Accidental finding = 2 pts	Accidental finding = 1 pt

Endoscopic findings at diagnosis	Exudative/hyperemic esophagus = 17 pts	Exudative/hyperemic esophagus = 10 pts
	Negative = 13 pts	Trachealization = 3 pts
	Trachealization = 0 pts	Stenosis/substenosis = 1 pt
	Stenosis/substenosis = 0 pt	Negative = 1 pt

Food allergy	20 pts	11 pts
	Profiline = 2 pts	Profiline = 5 pts
	PR-10 = 3 pts	LTP = 3 pts
	LTP = 3 pts	Milk = 7 pts
	Milk = 7 pts	Egg = 7 pts
	Egg = 12 pts	Tree nuts = 5 pts
	Tree nuts = 5 pts	Shellfish = 1 pt
	Shellfish = 1 pt	Wheat = 4 pts
	Wheat = 4 pts	Soy = 4 pts
	Soy = 3 pts	
	Fish = 1 pt	

Allergy to inhalants	18 pts	9 pts
	Grasses = 14 pts	Grasses = 7 pts
	Trees = 6 pts	Trees = 3 pts
	House dust mites = 9 pts	House dust mites = 5 pts
	Molds = 7 pts	Molds = 1 pt
	Animals = 10 pts	Animals = 2 pts
	Compositae = 2 pts	Compositae = 5 pts

Seasonality of symptoms	5 pts	4 pts
Pharmacological therapy at diagnosis	25 pts	11 pts
	Fluticasone+PPI = 24 pts	Fluticasone+PPI = 9 pts
	Only fluticasone = 1 pt	Only PPI = 1 pt
		Only fluticasone = 1 pt

Dietetic therapy	21 pts	11 pts
	SFED = 3 pts	Elimination diet = 11 pts
	Elimination diet = 18 pts	

Oral steroids	5 pts	6 pts

Endoscopic dilatation	2 pts	1 pt

Therapy in case of flare-up		Elimination diet = 5 pts
		Fluticasone+PPI = 9 pts
		Oral steroids = 5 pts

Maternal breastfeeding	17 pts	9 pts

Repeated use of antibiotic therapy during infancy	15 pts	10 pts

VAS at the diagnosis (mean)	7.93	8.66

VAS at the follow-up (mean)	1.73	3.26

Note: 4 patients underwent double therapies in case of refractory EoE.

**Table 2:** Characteristics of adult population (group B).

Variables	Responder patients (no. 15)	Refractory EoE patients (no. 20)
Age at the diagnosis (mean age)	32.7	39.4

Sex	12 M, 3 F	15 M, 5 F

Number of flare-ups (*n*, % pts)		1 = 4 pts
		2 = 8 pts
		3 = 1 pt
		4 = 5 pts
		8 = 1 pt
		15 = 1 pt

Diagnosis' delay from time of the symptom onset (mean in years)	4.96	5.55

Follow-up (mean in years)	4.66	3.0

Symptoms at the onset of EoE	Dysphagia = 8 pts	Esophageal bolus = 10 pts
	Esophageal bolus = 5 pts	Dysphagia = 8 pts
	Epigastric/abdominal pain = 5 pts	Epigastric/abdominal pain = 5 pts
	Vomit = 3 pts	Vomit = 1 pt
	Accidental finding = 2 pts	Accidental finding = 1 pt

Endoscopic findings at diagnosis	Exudative/hyperemic esophagus = 9 pts	Exudative/hyperemic esophagus = 18 pts
	Trachealization = 6 pts	Trachealization = 9 pts
	Negative = 5 pts	Stenosis/substenosis = 1 pt
	Stenosis/substenosis = 0 pt	Negative = 0 pt

Food allergy	12 pts	11 pts
	Profiline = 4 pts	Profiline = 3 pts
	PR-10 = 2 pts	PR-10 = 2 pts
	LTP = 7 pts	LTP = 1 pt
	Milk = 3 pts	Milk = 2 pts
	Egg = 4 pts	Egg = 3 pts
	Tree nuts = 1 pt	Tree nuts = 1 pt
	Shellfish = 3 pts	

Allergy to inhalants	10 pts	13 pts
	Grasses = 8 pts	Grasses = 12 pts
	Trees = 5 pts	Trees = 3 pts
	House dust mites = 5 pts	House dust mites = 6 pts
	Molds = 1 pt	Molds = 1 pt
	Animals = 4 pts	Animals = 5 pts
	Compositae = 2 pts	Compositae = 1 pt

Seasonality of symptoms	6 pts	12 pts

Pharmacological therapy at diagnosis	10 pts	18 pts
	Fluticasone+PPI = 8 pts	Fluticasone+PPI = 18 pts
	Oral steroids+PPI = 1 pt	
	Only PPI = 1 pt	

Dietetic therapy	14 pts	10 pts
	SFED = 2 pts	SFED = 3 pts
	Elimination diet = 12 pts	Elimination diet = 7 pts

Oral steroids	8 pts	3 pts

Endoscopic dilatation	0 pt	1 pt

Therapy in case of flare-up		Elimination diet = 4 pts
		Fluticasone+PPI = 16 pts

Maternal breastfeeding	12 pts	15 pts

Repeated use of antibiotic therapy during infancy	5 pts	14 pts

Tabagic habit	8 pts	9 pts

VAS at the diagnosis (mean)	8.66	7.7

VAS at the follow-up (mean)	2.0	2.7

**Table 3:** Pearson correlation in pediatric population (group A).

	Flare-ups	
	*r*	*p*
Years of follow-up	0.11	0.43
Age at diagnosis	0.02	0.88
Diagnosis' delay from time of the symptom onset	-0.17	0.26
Sex	-0.2	0.18
Food allergy	0.06	0.65
Allergy to inhalants	0	1
Symptoms at the onset of EoE	-0.08	0.5
Repeated use of antibiotic therapy during infancy	0.15	0.29
Seasonality of symptoms	0.11	0.44
Pharmacological therapy at diagnosis	-0.05	0.71
Dietetic therapy at diagnosis	-0.05	0.71
Maternal breastfeeding	0.01	0.91
VAS at follow-up	-0.37	**0.01**
VAS at diagnosis	-0.19	0.2

**Table 4:** Multivariate regression analysis in group A.

Variable	*β* ± SE	*p*
Years of follow-up	0.02 ± 0.04	0.63
Age at diagnosis	-0.01 ± 0.019	0.33
Diagnosis' delay from time of the symptom onset	-0.06 ± 0.05	0.28
VAS at follow-up	-0.1 ± 0.04	**0**.**02**
Sex	-0.32 ± 0.18	**0**.**05**
Food allergy	0.003 ± 0.21	0.98
Allergy to inhalants	-0.36 ± 0.64	0.57
Repeated use of antibiotic therapy during infancy	0.09 ± 0.14	0.49
Symptoms at the onset of EoE	-0.07 ± 0.07	0.27
Pharmacological therapy at diagnosis	0.20 ± 0.21	0.35
Dietetic therapy at diagnosis	-0.09 ± 0.15	0.55
Maternal breastfeeding	-0.05 ± 0.16	0.71

**Table 5:** Pearson correlation in adult population (group B).

	Flare-ups	
	*r*	*p*
Years of follow-up	0.39	**0**.**01**
Age at diagnosis	-0.25	0.13
Diagnosis' delay from time of the symptom onset	-0.05	0.76
Sex	-0.0	1
Food allergy	-0.26	0.13
Allergy to inhalants	0-0.01	0.92
Symptoms at the onset of EoE	-0.07	0.66
Repeated use of antibiotic therapy during infancy	0.36	**0**.**03**
Seasonality of symptoms	0.19	0.25
Pharmacological therapy at diagnosis	-0.32	0.26
Dietetic therapy at diagnosis	-0.31	0.06
Maternal breastfeeding	0.11	0.94
Tabagic habit	0.13	0.72
VAS at follow-up	-0.06	0.69
VAS at diagnosis	-0.19	0.2

**Table 6:** Multivariate regression analysis in group B.

Variable	*β* ± SE	*p*
Years of follow-up	0.08 ± 0.07	0.25
Age at diagnosis	-0.008 ± 0.007	0.24
Diagnosis' delay from time of the symptom onset	-0.025 ± 0.013	**0**.**04**
VAS at follow-up	-0.03 ± 0.07	0.61
Sex	-0.17 ± 0.17	0.35
Food allergy	0.48 ± 0.19	**0**.**01**
Allergy to inhalants	0.07 ± 0.80	0.92
Repeated use of antibiotic therapy during infancy	0.28 ± 0.19	**0**.**04**
Symptoms at the onset of EoE	0.07 ± 0.07	0.28
Pharmacological therapy at diagnosis	0.37 ± 0.24	0.13
Dietetic therapy at diagnosis	-0.21 ± 0.13	0.11
Maternal breastfeeding	-0.09 ± 0.14	0.5

## Data Availability

The data used to support the findings of this study are available from the corresponding author upon request.

## Abbreviations

EoE: Eosinophilic esophagitis
TSLP: Thymic stromal lymphopoietin
VAS: Visual analogue scale
HPFs: High-powered fields
NSAID: Nonsteroidal anti-inflammatory drugs.
